# A Rare Side Effect of Vaccine-Induced Thyrotoxicosis: A Case Report

**DOI:** 10.7759/cureus.96948

**Published:** 2025-11-16

**Authors:** Muhammad Munir, Adnan Abdullah, Gideon Mlawa, Balaji Murakonda, Zahid Khan

**Affiliations:** 1 Geriatrics, Queen’s Hospital, Romford, GBR; 2 Internal Medicine, Barking, Havering and Redbridge University Hospitals NHS Trust, Romford, GBR; 3 Internal Medicine and Diabetes and Endocrinology, Barking, Havering and Redbridge University Hospitals NHS Trust, Romford, GBR; 4 Care of the Elderly, Barking, Havering and Redbridge University Hospitals NHS Trust, Romford, GBR; 5 Cardiology, Queen’s Hospital, Romford, GBR

**Keywords:** autoimmune-like, covid-19, endocrine, graves' disease, hyperthyroid, hyperthyroid crisis, mrna-based vaccine, thyroid peroxidase antibodies, thyroid-stimulating hormone (tsh), vaccine-associated autoimmune thyroid disease

## Abstract

Graves’ disease is an autoimmune thyroid disorder driven by thyroid-stimulating hormone receptor antibodies (TRAb), resulting in hyperthyroidism. Autoimmune thyroid dysfunction has increasingly been reported following SARS-CoV-2 infection and, more recently, in association with COVID-19 vaccination. This case highlights a potential link between the ChAdOx1 nCoV-19 (AstraZeneca) vaccine and new-onset Graves’ disease. A 49-year-old man presented with a two-week history of palpitations, weight loss, diarrhea, and dyspnea. Symptoms began approximately two weeks after his second dose of the AstraZeneca COVID-19 vaccine. He had no prior thyroid history but had a background of Crohn’s disease in remission. Examination revealed tachycardia and a diffusely enlarged, non-tender thyroid gland. Laboratory findings demonstrated suppressed TSH (<0.01 mU/L), markedly elevated free thyroxine (>100 pmol/L), and raised TRAb (9.56 IU/L). Thyroid ultrasound showed diffusely enlarged lobes with heterogeneous echotexture and increased vascularity, consistent with Graves’ disease. The patient was treated with carbimazole, propranolol, and corticosteroids, with gradual clinical and biochemical improvement. At one-year follow-up, he remained euthyroid with TRAb levels reduced to 1.25 IU/L and no relapse of symptoms. Clinicians should consider vaccine-associated autoimmune thyroid disease in patients presenting with hyperthyroid symptoms shortly after COVID-19 vaccination. Early recognition, confirmation through TRAb testing, and appropriate management are critical. Long-term follow-up is essential to monitor for relapse or remission in post-vaccination Graves’ disease.

## Introduction

Graves’ disease is an autoimmune disorder characterised by the production of thyroid-stimulating hormone receptor antibodies (TRAb), which activate thyroid-stimulating hormone (TSH) receptors on thyroid follicular cells, leading to excessive synthesis and secretion of thyroxine (T4) and triiodothyronine (T3) [1].

Since the emergence of the coronavirus disease 2019 (COVID-19) pandemic, the causative virus, severe acute respiratory syndrome coronavirus 2 (SARS-CoV-2), has rapidly spread worldwide, resulting in substantial morbidity and mortality. As of June 2025, the World Health Organization (WHO) reported a cumulative total of 778,050,175 confirmed cases and 7,096,935 deaths globally, underscoring the profound impact of the pandemic on global public health [2].

Following the widespread administration of COVID-19 messenger RNA (mRNA) vaccines, a range of adverse events has been documented. These include cardiovascular complications, such as acute coronary syndrome (ACS) and myocarditis, endocrine disorders including Graves’ disease, Graves’ orbitopathy, subacute thyroiditis, and silent thyroiditis, as well as autoimmune phenomena such as Guillain-Barré syndrome, autoimmune haemolytic anaemia, autoimmune thrombotic thrombocytopenic purpura, and immune thrombocytopenic purpura. Notably, COVID-19 infection itself has also been associated with thyroid dysfunction, particularly subacute thyroiditis [3-14].

Several case reports have suggested that SARS-CoV-2 infection may trigger subacute thyroiditis through immune-mediated mechanisms [4,5]. Similarly, post-vaccination thyroid autoimmunity, including new-onset Graves’ disease and thyroiditis, has been reported following administration of mRNA-based vaccines [6]. These phenomena have been discussed within the framework of autoimmune/inflammatory syndrome induced by adjuvants (ASIA) [8]. Case reports have described the development of Graves’ disease after SARS-CoV-2 mRNA vaccination, and others have documented exacerbation of subclinical hyperthyroidism in temporal association with vaccination [8,9].

Mechanistically, cross-reactivity between SARS-CoV-2 proteins and human tissue antigens has been proposed as a potential driver of post-vaccination autoimmunity [10]. Furthermore, new-onset autoimmune conditions affecting multiple organ systems have been reported following COVID-19 vaccination [11]. An increased incidence of Graves’ disease has also been observed during the COVID-19 pandemic, with emerging evidence suggesting distinct clinical characteristics in post-vaccination, early-onset Graves’ disease [12,13]. A recent review summarised various forms of thyroiditis following COVID-19 vaccination, further supporting the link between immune activation and thyroid autoimmunity [14].

## Case presentation

A 49-year-old man presented to the Accident and Emergency Department with complaints of shortness of breath, palpitations, unintentional weight loss, and diarrhoea over the preceding two weeks. His past medical history was notable for Crohn’s disease, which was in remission at the time of presentation. Notably, he had received his second dose of the SARS-CoV-2 (AstraZeneca) vaccine two weeks before the onset of symptoms. He denied any recent iodine exposure or use of amiodarone, and there was no known family history of thyroid disease.

On examination, the patient appeared anxious and tachycardic, with a pulse rate of 118 beats per minute and a regular rhythm. He was afebrile, normotensive, and clinically euthyroid, with no evidence of thyroid eye disease. Examination of the neck revealed a diffusely enlarged, non-tender thyroid gland without bruit or nodularity. Cardiovascular and respiratory examinations were otherwise unremarkable. Initial laboratory investigations on admission revealed biochemical evidence of thyrotoxicosis, with a suppressed TSH level (<0.01 mU/L) and markedly elevated free thyroxine (free T4 >100 pmol/L). TSH receptor antibodies were significantly elevated at 9.56 IU/L, confirming the diagnosis of Graves’ disease. Haemoglobin, white cell count, urea, and creatinine were within normal limits. The relevant biochemical results are summarised in Table 1.

**Table 1:** Initial laboratory investigations demonstrating biochemically severe thyrotoxicosis, evidenced by suppressed TSH, markedly elevated free T4, and positive TSH receptor antibodies confirming Graves’ disease TSH - Thyroid-stimulating hormone

Test	Result	Normal Range
TSH	<0.01 mU/L	0.27-4.2 mU/L
Free T4	>100 pmol/L	11.9-21.6 pmol/L
TSH Receptor Antibodies	9.56 IU/L	0-0.4 IU/L
Hemoglobin	124.0 g/L	133-173 g/L
White Cell Count	6.0 x 10^9^/L	3.8-11.0 x 10^9^/L
Platelet Count	209 x 10^9^/L	150-400 x 10^9^/L
Sodium	140 mmol/L	133-146 mmol/L
Potassium	4.8 mmol/L	3.5-5.3 mmol/L
Urea	9.9 mmol/L	2.5-7.8 mmol/L
Creatinine	65.0 umol/L	59-104 umol/L
ALBMUIN	33.0 g/L	35-50 g/L
Corrected Calcium	2.64 mmol/L	2.20-2.60 mmol/L

**Figure 1:**
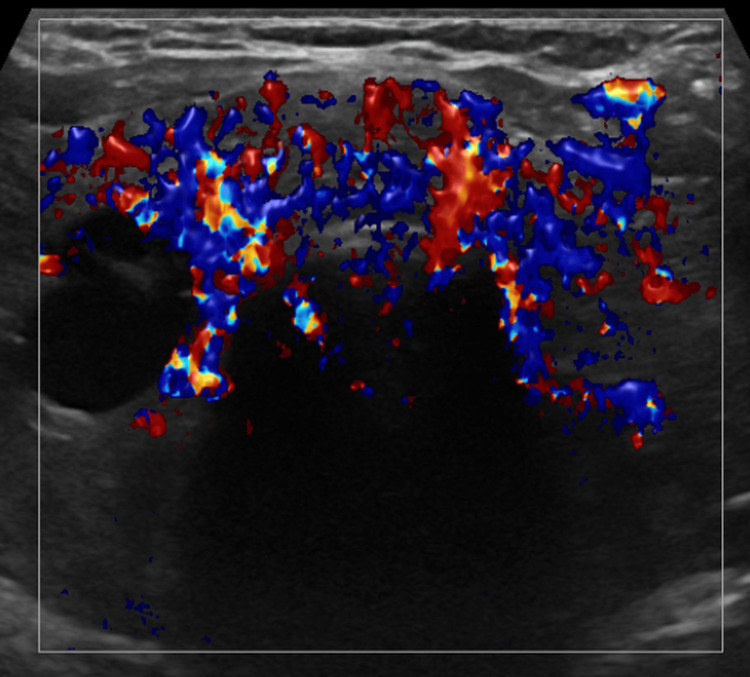
Ultrasound of the thyroid showing diffusely enlarged and heterogeneous thyroid lobes with increased vascularity, consistent with Graves’ disease

Ultrasonography (USG) of the thyroid demonstrated diffuse enlargement of both lobes with a heterogeneous echotexture and markedly increased vascularity on colour Doppler imaging, consistent with Graves’ disease (Figure 1). No focal nodules or cystic lesions were identified.

The patient was promptly treated with intravenous hydrocortisone, initiated due to concern for possible thyroid storm or severe thyrotoxicosis. He was subsequently commenced on carbimazole 30 mg daily to inhibit thyroid hormone synthesis and propranolol 40 mg three times daily to achieve symptomatic control of adrenergic manifestations, including tachycardia and palpitations. Supportive measures were provided, including adequate hydration and close monitoring of cardiovascular status and biochemical parameters.

The patient’s symptoms gradually improved over the following days, with resolution of palpitations and improvement in gastrointestinal symptoms. He was discharged with continued oral carbimazole and propranolol therapy, along with tapering of corticosteroids as clinically indicated. Arrangements were made for early follow-up in the Endocrinology Outpatient Hot Clinic for ongoing assessment, thyroid function monitoring, and titration of antithyroid therapy. Carbimazole 30 mg once daily and propranolol 40 mg thrice daily were initiated. Carbimazole was gradually down-titrated based on serial thyroid function results. The dose was initially reduced to 10 mg once daily, with propranolol tapered to 10 mg twice daily. Over the subsequent months, carbimazole was further decreased to 5 mg on alternate days, which was maintained for six months before being discontinued.

Case progression

Follow-up thyroid function tests performed at three months and one year demonstrated progressive biochemical improvement, corresponding with marked clinical recovery. The patient achieved a sustained euthyroid state, with resolution of palpitations, weight loss, and gastrointestinal symptoms.

The most recent laboratory evaluation showed TSH 1.00 mU/L, free T4 17 pmol/L, and a decline in TSH receptor antibody titre to 1.25 IU/L, indicating remission of autoimmune activity.

**Figure 2:**
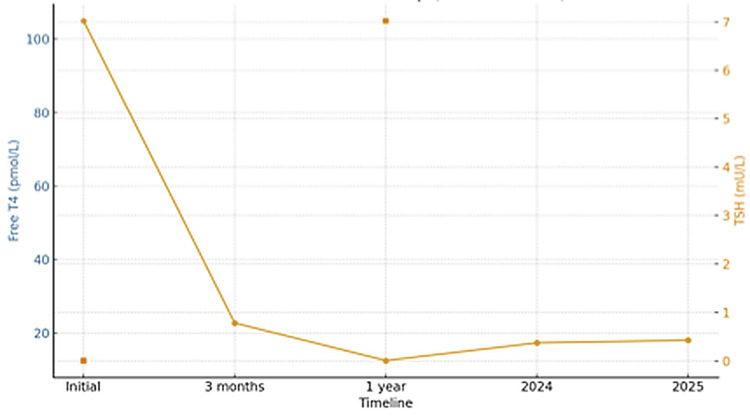
Timeline demonstrating the patient’s treatment course and progressive biochemical improvement over time TSH - Thyroid-stimulating hormone

The patient has since remained clinically and biochemically euthyroid, with no evidence of relapse following cessation of therapy (Figure 2).

## Discussion

This case describes a 45-year-old man who developed Graves’ disease following his second dose of a COVID-19 vaccine [8]. The patient demonstrated a favourable clinical and biochemical response to thiamazole therapy, with a progressive decline in TRAb levels and resolution of hyperthyroid symptoms. Recent data indicate that new-onset Graves’ disease occurring within four weeks of vaccination tends to present in older individuals (median age: 51 years versus 35 years). These cases also appear to be more frequent in men (40.0% versus 13.6%). The distribution of reported cases according to vaccine dose was 25% after the first, 65% after the second, and 10% after the third dose [13]. The demographic profile and temporal relationship in this patient are consistent with these observations.

Early therapeutic responses to antithyroid medication have generally been favourable. Approximately 40% of affected individuals achieve negative TRAb titres (≤1.75 IU/L) within three months of treatment initiation. This suggests that the vaccine-related autoimmune response may be transient or self-limiting [13].

However, a high TRAb titre at diagnosis (>12 IU/L) or persistent positivity at treatment cessation (>1.5 IU/L) is associated with an increased risk of relapse. Most recurrences occur within two years, warranting close clinical and biochemical follow-up [15].

Therefore, despite an initially favourable response, treatment discontinuation should be approached with caution [16]. Serial TRAb measurements may provide valuable insight into disease progression following vaccination.

The development of Graves’ disease after COVID-19 vaccination is believed to arise primarily through autoimmune mechanisms involving TRAb production [6-15].

ASIA has been described following vaccinations against human papillomavirus, influenza, and hepatitis B virus. Although vaccine adjuvants enhance immunogenicity, they may also provoke aberrant immune activation in predisposed individuals. Aluminium-based adjuvants, widely used in vaccines such as those for human papillomavirus and hepatitis B, have been implicated in ASIA pathogenesis [6,7,14,17,18].

mRNA vaccines utilise lipid nanoparticles (LNPs) as delivery systems [19]. These LNPs induce robust inflammatory cytokine responses, notably interleukin (IL)-1β and IL-6, potentially contributing to ASIA [19,20].

Compared with MF59-like adjuvants such as AddaVax, LNPs elicit markedly higher levels of IL-1β (up to 22-fold) and IL-6 (≥12-fold) and generate stronger antibody responses [19]. Thyroid tissue expresses angiotensin-converting enzyme 2 (ACE2), which serves as a binding site for the SARS-CoV-2 spike protein [6]. The interaction between the spike protein and ACE2 results in ACE2 downregulation and the release of IL-1β and IL-6, both of which promote inflammation.

Elevated levels of these cytokines may further enhance autoimmunity through activation of T helper 17 cells and suppression of regulatory T cells [11]. Genetic susceptibility to ASIA has also been linked to specific HLA-DRB1 haplotypes [18].

Molecular mimicry represents another plausible mechanism of post-vaccination autoimmunity [10]. Homology between amino acid sequences of thyroid peroxidase (TPO) and the SARS-CoV-2 spike protein has been demonstrated, suggesting potential cross-reactivity [11]. Longitudinal studies among Japanese healthcare workers have reported gradual increases in mean TRAb levels with repeated mRNA vaccination [17]. Although TPO antibodies were not assessed in the present case, simultaneous measurement of TPO and TRAb may yield a more comprehensive evaluation of post-vaccination thyroid autoimmunity.

Risk factors for Graves’ disease include genetic predisposition and the interplay of endogenous and environmental factors, such as oestrogen exposure, X-chromosome inactivation, smoking, iodine excess, selenium or vitamin D deficiency, and occupational exposure to agents such as Agent Orange. Genetic susceptibility linked to HLA-B35, HLA-C04, and HLA-A*11 alleles has been associated with SARS-CoV-2 vaccine-induced subacute thyroiditis [14]. Recurrence of Graves’ disease following vaccination has also been observed among patients with a prior history of the condition. Symptoms, including palpitations, weight loss, and tremor, have typically developed after the second vaccine dose. Given the absence of other precipitating factors and the temporal relationship between vaccination and symptom onset, a causal association between vaccination and exacerbation of hyperthyroidism is strongly suggested.

Graves’ disease remains a relatively rare adverse event compared with myocarditis or acute coronary syndrome [3,8]. It may therefore be underrecognized. In the present case, the patient was initially referred with presumed exacerbation of Crohn’s disease, delaying the diagnosis of thyrotoxicosis [8].

The onset of Graves’ disease symptoms following vaccination has been reported to range between two and 20 days. Clinicians should maintain a high index of suspicion for hyperthyroidism in patients presenting with sinus tachycardia, absence of ST-segment changes on electrocardiogram, and normal cardiac biomarkers after recent COVID-19 vaccination [6].

## Conclusions

This case describes a male patient who developed Graves’ disease and remained positive for TRAb one year after receiving an mRNA-based COVID-19 vaccine. In individuals presenting with symptoms suggestive of thyrotoxicosis following vaccination, evaluation of thyroid function, including measurements of free thyroxine (FT4) and TSH, is essential to identify potential thyroid-related adverse effects. The long-term trajectory of post-vaccination Graves’ disease remains uncertain, underscoring the importance of ongoing clinical and biochemical monitoring to guide management and assess disease remission.
